# Influence of Exosomes on Astrocytes in the Pre-Metastatic Niche of Lung Cancer Brain Metastases

**DOI:** 10.1186/s12575-023-00192-4

**Published:** 2023-03-01

**Authors:** Lingyun Ye, Yinfei Wu, Juan Zhou, Mengqing Xie, Zhemin Zhang, Chunxia Su

**Affiliations:** 1 Department of Oncology, Shanghai Pulmonary Hospital & Thoracic Cancer Institute, Tongji University School of Medicine, No. 507, Zheng Min Road, Shanghai, 200433 China; 2 Department of Respiratory Intervention, The Affiliated Cancer Hospital of Zhengzhou University & Henan Cancer Hospital, No.127, Dongming Road, Jinshui District, Zhengzhou, 450008 China

**Keywords:** Exosome, Astrocyte, Pre-metastatic niche, Lung cancer brain metastases

## Abstract

**Background:**

Lung cancer is the most common cause of cancer-related death globally. There are several reasons for this high mortality rate, including metastasis to multiple organs, especially the brain. Exosomes play a pivotal role in tumor metastasis by remodeling the microenvironment of remote target organs and promoting the pre-metastatic niche’s formation. Since astrocytes are indispensable for maintaining the homeostasis of brain microenvironment, it’s of great interest to explore the influence of lung cancer cell-derived exosomes on astrocytes to further understand the mechanism of lung cancer brain metastasis.

**Results:**

Twenty four h after co-culture of H1299 cell-derived exosomes and SVG P12 cells, the viability of astrocytes decreased and the apoptosis increased. The levels of cytokines in the supernatant including GROα/CXCL1, IFN-γ, IL-3, IL-5, IL-15, LIF, M-CSF, NGF, PDGF, and VEGF were significantly enhanced, while IL-7 secretion was significantly reduced. Meanwhile, apoptosis-related proteins MAP2K1, TUBA1C, RELA, and CASP6 were up-regulated. And the differentially expressed proteins were involved in regulating metabolic pathways.

**Conclusion:**

Exosomes of H1299 could induce apoptosis of astrocytes as well as promote their secretion of cytokines that were conducive to the formation of the inflammatory microenvironment and immunosuppressive microenvironment, and affect their metabolic pathways, thus facilitating the formation of pre-metastatic niche in lung cancer brain metastases.

## Introduction

The gradually increasing incidence rate of brain metastasis in lung cancer seriously affects patients’ quality of life and leads to poor prognosis [1, 2]. Up to now, the central nervous system has been considered as the “refuge” of brain metastases. However, due to the blood-brain barrier (BBB), most drugs do not reach an effective therapeutic concentration in the brain, which could affect the therapeutic effect of brain metastases [3].

Accumulating evidence has shown that primary tumors induce a microenvironment conducive to metastasis called pre-metastatic niche (PMN) at a specific organ and tissue site prior to metastasis [4, 5]. An essential factor in forming PMN in the brain lies in destroying of the BBB [6]. During the brain metastasis process, tumor cells separated from the primary tumor enter the systemic circulation, stop at the branch of cerebral micro vessels, and then pass through the damaged BBB to reach the brain parenchyma [7].

The BBB is composed of endothelial cells, pericytes, and astrocytes [8]. Astrocytes are the most widely distributed and abundant glial cells in the brain, which are involved in maintaining BBB characteristics and the stability of the brain microenvironment [9]. With a mouse model of lung cancer brain metastasis, we have previously investigated the change of BBB structure in brain metastases and found that astrocytes distributed and surrounded the blood vessels in the normal brain or the tumor border but not the inner metastatic lesions [10]. It seems that loss of astrocytes will destroy BBB and promote the formation of PMN, therefore further studies are required to clarify the underlying mechanisms.

In recent years, tumor cell-derived exosomes have attracted extensive attention as intercellular communication materials. They play a significant role in mediating the formation of PMN [11, 12]. Tumor cell-derived exosomes usually carry important signal molecules, such as miRNA, which can reshape the tumor microenvironment by regulating immune response and angiogenesis, thus participating in the metastasis of tumor cells [13]. In the brain PMN formation process, the exosomes secreted by tumor cells play the role of “former sentry” since they reach the brain tissue ahead of circulating tumor cells (CTCs) and exert the function of compromising the BBB integrity to accelerate tumor cells to pass through BBB. For example, Tominaga et al. showed that the exosomes derived from breast cancer cells could transfer miR-181c to brain endothelial cells and change the expression of tight junction proteins as well as the position of actin filaments, thereby disrupting the tight junction between endothelial cells and leading to increased BBB permeability [14]. Treatment of astrocytes with exosomes released from precursor B acute lymphoblastic leukemia embryo cells leads to the upregulation of vascular endothelial growth factor (VEGF), a vital factor that mediates leukemia infiltration into the central nervous system [15]. However, the effect of lung cancer cell-derived exosomes on astrocytes during the formation of brain PMN in lung cancer brain metastasis had not been reported so far.

Therefore, we co-cultured exosomes of non-small cell lung cancer (NSCLC) cell line H1299 with human astrocytes to explore their effects on astrocytes and reveal the role of lung cancer cell-derived exosomes on the formation of brain PMN. Overall, our study provides a new theoretical basis for lung cancer brain metastasis.

## Results

### Identification of Exosomes

**Fig. 1:**
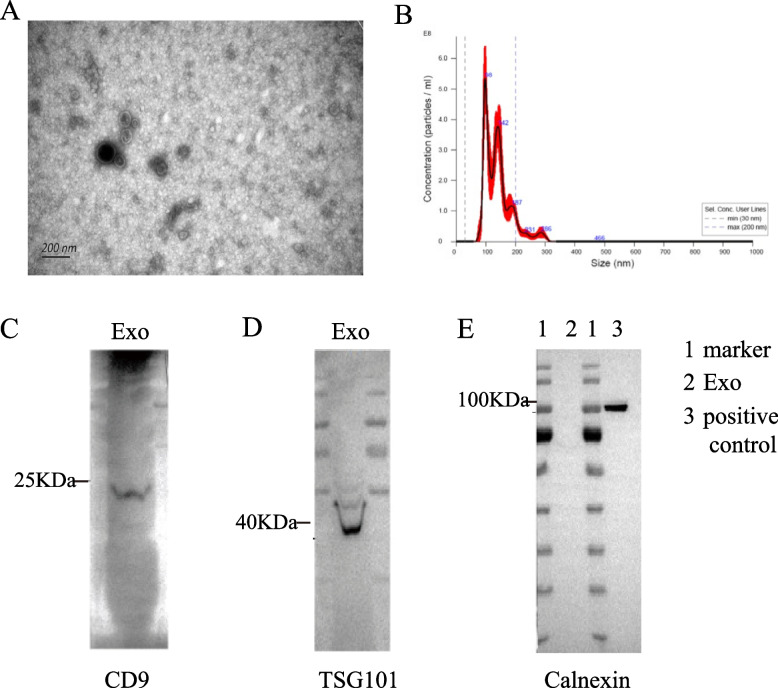
Identification of exosomes extracted from the supernatant of H1299 cells. **A** Electron microscope was used to detect the morphology of exosomes. It could be seen that exosomes were saucer-like double concave disks. **B** The size of exosomes was analyzed by NTA particle size detection. The result showed that the highest exosome diameter was about 98 nm. **C** Western Blot was conducted to detect the expression of exosomes-related protein marker. The results showed that CD9 and TSG 101 proteins were positively expressed on exosomes, while Calnexin proteins were negatively expressed

As shown in Fig. 1A, the exosomes of H1299 cell line showed a typical saucer-like double concave disc with a distinct membrane, and the size was about 100 nm under electron microscopy. NTA particle size result showed that the maximum exosome particle diameter of H1299 cells was about 98 nm (Fig. 1B). Western Blot showed that the extracted exosome-positive protein markers CD9, Tsg101, and CD63 were expressed, while the negative-protein marker Calnexin did not expressed (Fig. 1C, D, E). This suggested that the quality of extracted exosomes was up to the standard and could be used in subsequent experiments.

### Effect of H1299 Cell-Derived Exosomes on the Viability of Astrocytes

**Fig. 2:**
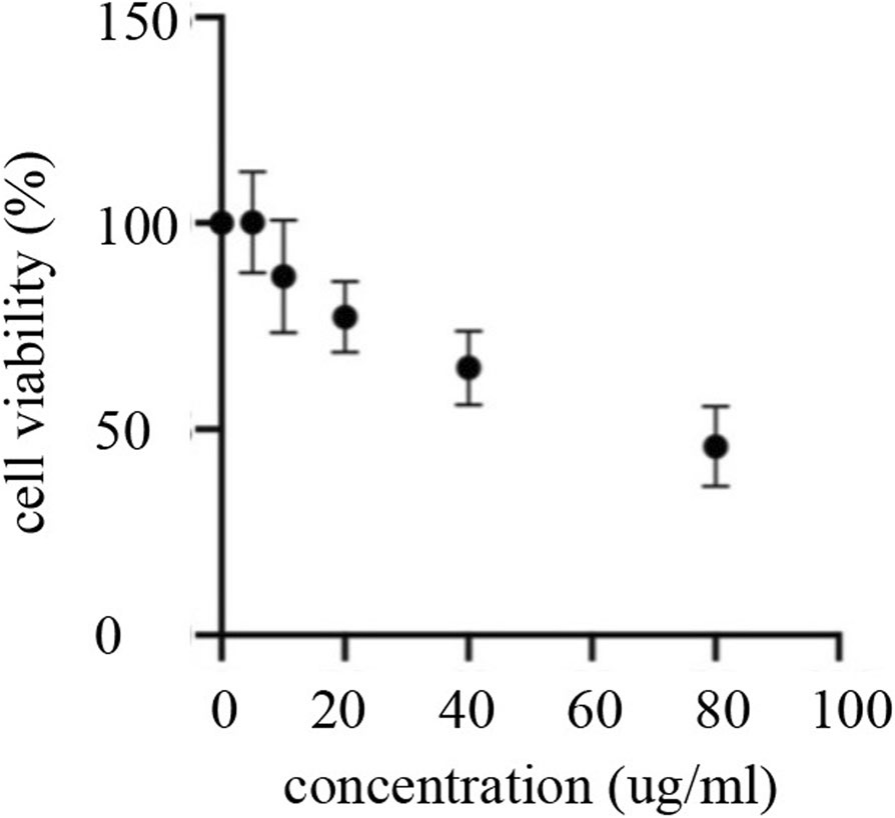
Effects of H1299 cell-derived exosomes on the viability of SVG P12 cells. Different concentrations of exosomes were co-cultured with SVG P12 cells for 24 h, and the cell viability was detected by CCK8 assay. The results showed that H1299 cell-derived exosomes could reduce the cell viability of astrocytes, and the cell viability was about 50% of that of the control group when the exosome concentration was 60μg/ml

H1299 cell-derived exosomes with different concentrations were added into the medium of SVG P12 cells and co-cultured for 24 hours. The viability of astrocytes was observed by the CCK8 experiment. As shown in Fig. 2, the activity of SVG P12 cells gradually declined with the increase of exosome concentration, suggesting that tumor-cell-derived exosomes could affect the activity of astrocytes. And when the concentration of exosomes reached 60 μg/ml, the viability of SVG P12 cells decreased by about 50%.

### Edu/Hoechst 33342 Experiment Was Conducted to Observe the Effect of Exosomes on Apoptosis of SVG P12 Cells

**Fig. 3:**
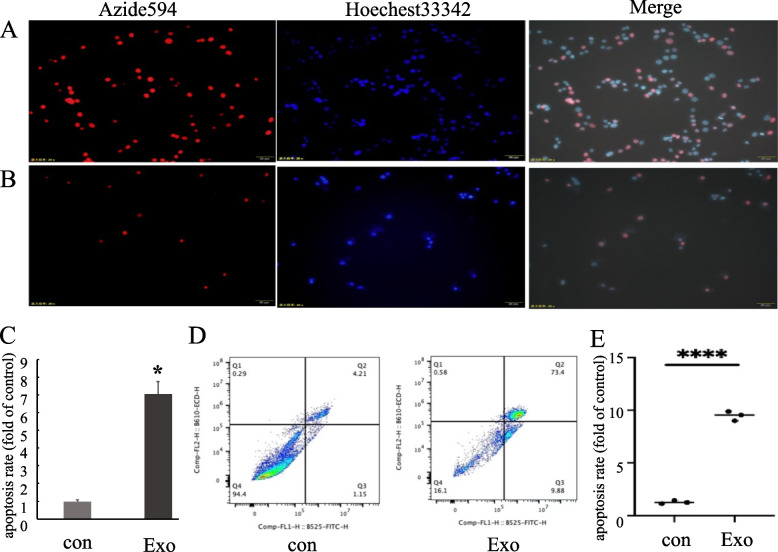
Effects of H1299 cell-derived exosomes on apoptosis of SVG P12 cells. **A** Edu/Hoechst33342 results of SVG P12 cells in the control group (SVG P12 cells without H1299 cell-derived exosomes). **B** Edu/Hoechst33342 results of SVG P12 cells after co-culturing with H1299 cell-derived exosomes for 24 h. **C** Statistical analysis of Edu/Hoechst33342 results (*p* < 0.05). **D** Flow cytometry results of SVG P12 cells in each group. **E** Statistical analysis of flow cytometry results (*p* < 0.0001). (con: SVG P12 cells without H1299 cell-derived exosomes. Exo: SVG P12 cells co-cultured with H1299 cell-derived exosomes for 24 h)

We co-cultured 60 μg/ml exosomes of H1299 cells with SVG P12 cells for 24 hours to detect the apoptosis of SVG P12 cells by Edu/Hoechst 33342 assay. From Fig. 3A-C we could see that the apoptosis of SVG P12 cells aggravated when H1299 cell-derived exosomes were added to the SVG P12 cell culture medium compared to the control group.

### Detection of Apoptosis of SVG P12 Cells co-Cultured with Exosomes by Flow Cytometry

To further explore the influence of exosomes extracted from H1299 cells on SVG P12, we used flow cytometry to measure the apoptosis of SVG P12 cells treated with H1299 cell-derived exosomes (the concentration was 60 μg/ml) for 24 h. The results showed that compared with the control group, the early and late apoptotic rates of the treatment group were significantly increased (Fig. 3D, E).

### Effect of H1299 Cell-Derived Exosomes on Cytokines Secreted by SVG12 Cells

**Fig. 4:**
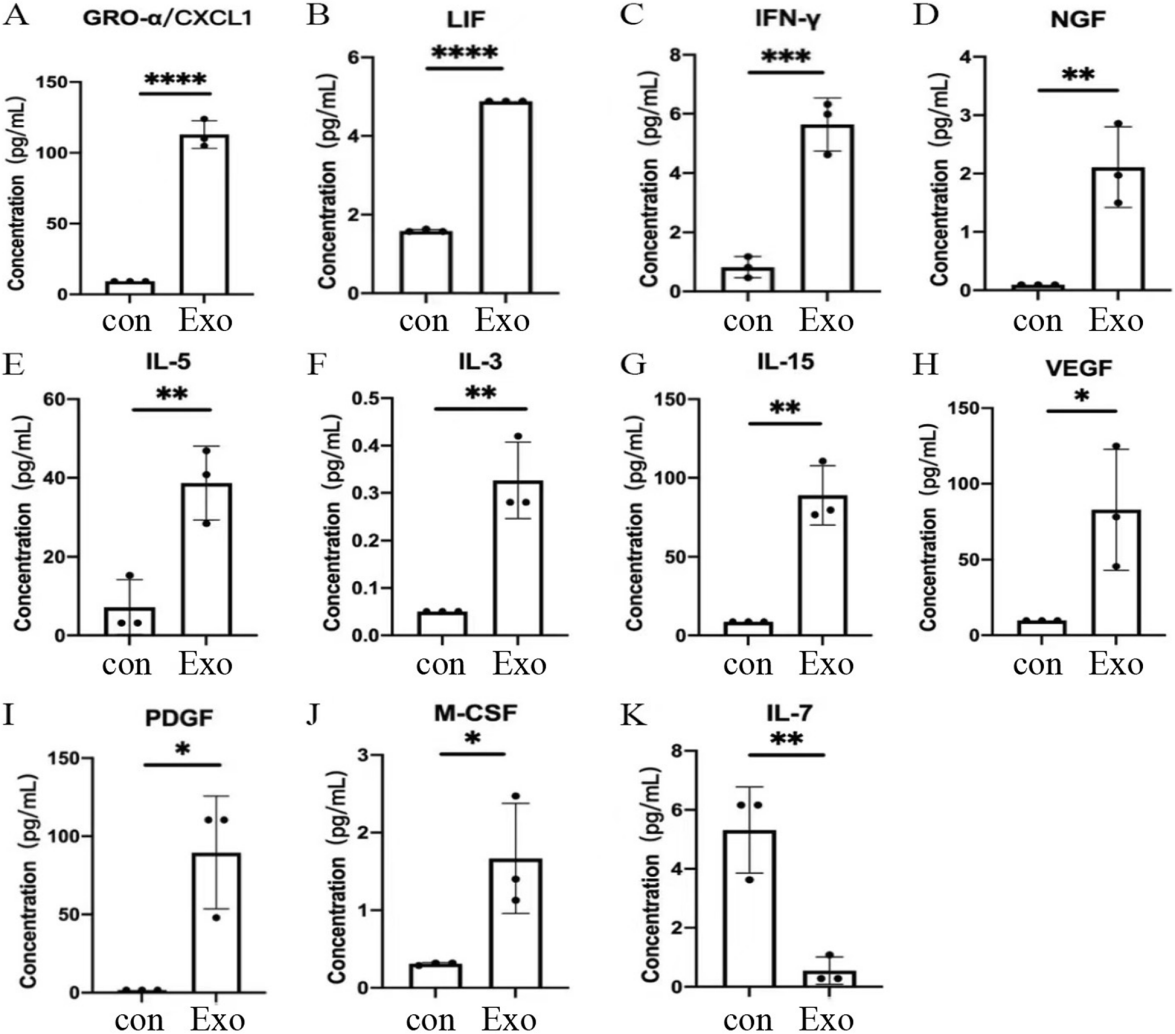
The most differentially secreted cytokines in the supernatant of SVG P12 cells. Detection of cytokines secreted by SVG P12 cells in each group by Luminex liquid suspension chip. Con: Cytokines secreted in the supernatant of SVG P12 cells cultured for 24 h without H1299 cell-derived exosomes. Exo: Cytokines secreted in the supernatant of SVG P12 cells after co-culturing with H1299 cell-derived exosomes (60 μg/ml) for 24 h

After co-culturing H1299 cell-derived exosomes with SVG P12 cells for 24 h, we detected the cytokines secreted by SVG P12 cells through the Luminex liquid suspension chip. It can be seen from Fig. 4, the cytokines secreted by SVG P12 cells were significantly changed after exosomes treatment. GROα/CXCL1, IFN-γ, IL-3, IL-5, IL-15, LIF, M-CSF, NGF, PDGF, and VEGF secreted by SVG P12 cells were significantly up-regulated, while IL-7 secretion was significantly reduced.

### Effect of H1299 Cell-Derived Exosomes on the Protein Expression of SVG12 Cells

Through proteomic analysis, we further explored the impact of H1299 cell-derived exosomes on the protein expression of SVG P12 cells. The control group was SVG P12 cells without exosomes treatment, and the experimental group was SVG P12 cells co-cultured with exosomes of H1299 cells for 24 h. The differences in protein expression between the two groups were compared.

**Fig. 5:**
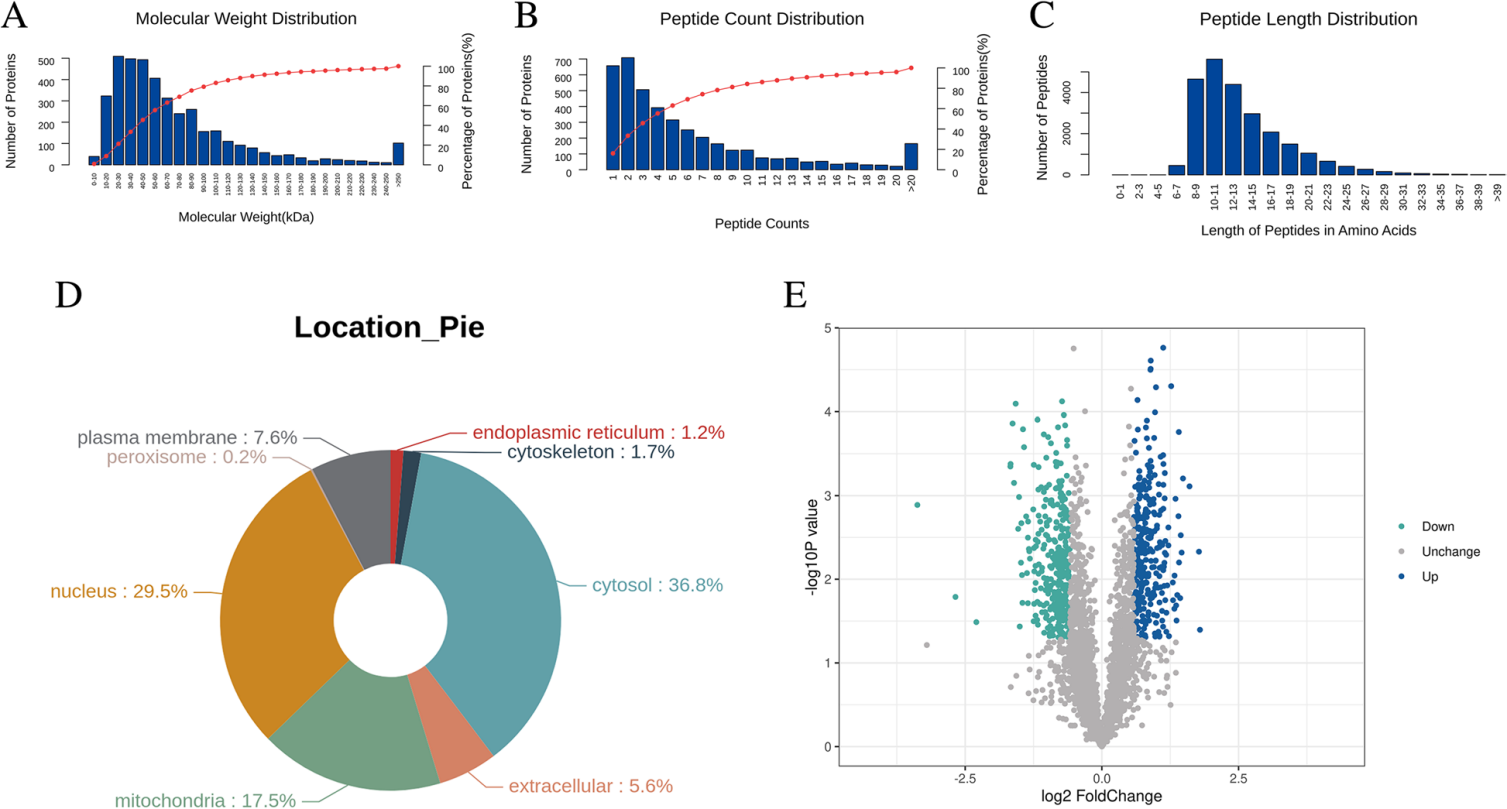
Characteristics of differentially expressed proteins in SVG P12 cells. Label-free quantitative proteomics was used to analyze the differentially expressed proteins. **A** Relative molecular weight distribution of differentially expressed proteins in SVG P12 cells. **B** Peptide count distribution of differentially expressed proteins in SVG P12 cells. **C** Peptide length distribution of differentially expressed proteins in SVG P12 cells. **D** Classification map of subcellular localization of differentially expressed proteins in SVG P12 cells. **E** Volcanic map of differentially expressed proteins in SVG P12 cells. Volcanic map was drawn by protein expression difference multiple and *P* value to show the difference between the two groups of samples. The blue dots in the figure were up-regulated proteins, the cyan dots were down-regulated proteins, and the gray black dots were non-differentially expressed proteins. The abscissa was the difference multiple, and the ordinate was the *P* value

Based on the relative molecular weight and peptide number distribution of differentially expressed proteins, almost 80% of the differentially expressed proteins had a molecular weight within 100 kDa (Fig. 5A), 60% of the protein peptides were less than 4 (Fig. 5B), and most of the peptides were distributed in 7–20 amino acids (Fig. 5C). Subcellular localization analysis showed that 36.8% of differentially expressed proteins were located in the cytoplasm, 29.5% in the nucleus, and 17.5% in mitochondria (Fig. 5D). Compared with the control group, 312 proteins were significantly overexpressed in the experimental group, and the first 9 proteins owing the most significant up-regulated changes were OSTF1, GCLC, ADH5, NT5C, EFL1, ATG4B, ACTC1, KLC3, and TBC1D1. The expression of 347 proteins were significantly decreased, among which GLS, ITGB7, TMSB10, MMUT, SQOR, OGDHL, PCK2, CPOX, CPD were the most significantly down-regulated proteins (Fig. 5E).

**Fig. 6:**
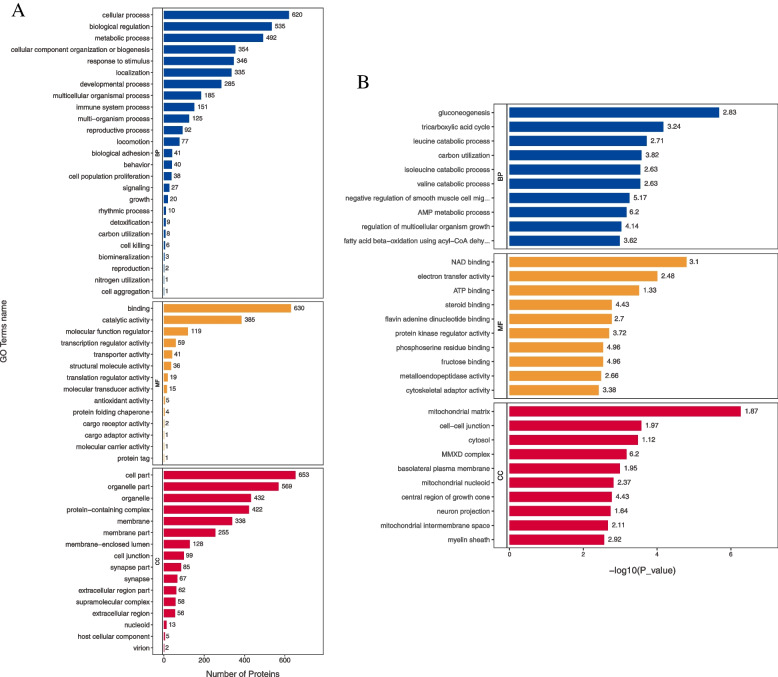
GO analysis of differentially expressed proteins in SVG P12 cells. **A** Results of GO function annotation of differentially expressed proteins in SVG P12 cells. The vertical axis showed the name of each GO function, and the value in horizontal axis was the number of differentially expressed proteins contained in that function. **B** Results of GO enrichment analysis of differentially expressed proteins in SVG P12 cells. The name of each GO function was displayed in the vertical axis, and the value in horizontal axis was the *P* value after -log10 conversion. Different color represented different GO function classification, and the number on the column was the enrichment factor

**Fig. 7:**
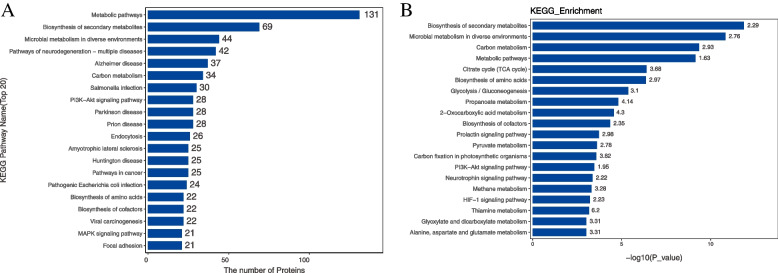
KEGG pathway analysis of differentially expressed proteins in SVG P12 cells. **A** Results of KEGG pathway annotation of differentially expressed proteins in SVG P12 cells. The vertical axis showed the name of each KEGG pathway, and the value in horizontal axis was the number of differentially expressed proteins contained in that pathway. **B** Results of KEGG pathway enrichment analysis of differentially expressed proteins in SVG P12 cells. The name of each KEGG pathway was displayed in the vertical axis, and the value in horizontal axis was the *P* value after -log10 conversion. The number on the column was the enrichment factor

Blast2Go software was used to annotate the GO functions of all differentially expressed proteins. The results showed that the biological processes involved in were mainly related to metabolism, including gluconeogenesis, tricarboxylic acid cycle, leucine catabolic process, carbon utilization, isoleucine catabolic process, valine catabolic process, AMP metabolic process, and fatty acid beta-oxidation. Molecular functions related include NAD binding, electron transfer activity, ATP binding, and protein kinase regulator activity. And the differentially expressed proteins were associated with a variety of cell components. GO enrichment analysis also showed that most of them participated in the metabolic process (Fig. 6). KEGG pathway annotation and KEGG enrichment analysis showed that differentially expressed proteins could participate in metabolic pathways regulation, biosynthesis of secondary metabolites, pathways of neurodegeneration-multiple disease, endocytosis, PI3K-Akt signaling pathway, MAPK signaling pathway, and so on (Fig. 7).

## Discussion

Previous studies have found that prior to metastasis, the primary tumor can reshape the microenvironment in a specific tissue and induce the formation of a pre-metastatic niche (PMN), which offers a valuable opportunity for the adaptation and survival of the primary tumor cells, enabling them to proliferate and obtain conditions for colonization in the target organ for subsequent metastasis [16]. The earliest event of PMN formation is that the primary tumor cells secrete various soluble factors and exosomes to regulate the information transmission from local to distant areas, thus changing the local microenvironment [17, 18]. In lung cancer brain metastasis, the prerequisite for brain PMN formation lies in the destruction of BBB.

As an essential component of BBB, astrocytes are indispensable in the immune microenvironment for brain metastasis of lung cancer. Valiente et al. proposed that astrocytes could lead to tumor cell death in the early stage of tumor metastasis. Therefore, tumor cells have to acquire properties that block apoptosis in order to facilitate metastasis to the brain [19]. On the other hand, a growing number of studies have also found that astrocytes participate in the cascade steps of tumor metastasis, including colonization and growth [20]. By activating astrocytes, tumor cells adapt to the new microenvironment and begin to colonize and migrate. It has been reported that brain metastatic tumor cells could trigger the transformation of astrocyte phenotype through the IL-1β-mediated NF-κB pathway to induce c-Met activation in tumor cells and promote their survival and growth [21]. Tumor cells could also activate cGAMP in STAT1 and NF-κB signaling pathways to form gap junctions with astrocytes and stimulate the latter to produce IFN-γ and other cytokines, which in turn support tumor cell growth [22]. In previous animal experiments, we found a decrease in the number of astrocytes in the brain metastasis of lung cancer compared with normal brain tissue [10]. This study proved for the first time that the exosomes secreted by lung cancer cell line H1299 could significantly change the expression of apoptosis related proteins MAP2K1, TUBA1C, RELA, and CASP6, thus promoting apoptosis of human astrocyte line SVG P12 cells. In conclusion, lung cancer cells promote astrocyte apoptosis through their secreted exosomes to enable the formation of brain PMN.

It has been found that cytokines secreted by tumor cells and host stromal cells can promote the formation of the local inflammatory microenvironment and immune inhibitory microenvironment. This microenvironment is the fundamental factor for the construction of PMN and could further promote the dynamic evolution of PMN and the subsequent invasion of tumor cells to target organs [23, 24]. We found that the cytokines secreted by astrocytes were significantly altered after treatment with tumor-derived exosomes. GROα/CXCL1, IFN-γ, IL-3, IL-5, IL-15, LIF, M-CSF, NGF, PDGF, and VEGF were significantly increased, while the level of IL-7 was significantly decreased. Most of these cytokines are involved in stimulating or recruiting the proliferation and aggregation of other immune cells. Yan Zhou et al. found that GRO-α could enhance the migration and infiltration ability of glioma cells [25]. LIF could induce nerve connections, stimulate astrocytes, and increase BBB permeability [26]. Zhezhi Deng et al. found that VEGF secreted by astrocytes could increase cerebral microvascular permeability [27]. Besides, astrocyte-produced IL-7 could promote the differentiation of monocytes into macrophages, induce the expression of MHC-I and II genes, and activate CD4+ and CD8+ T lymphocytes [28]. However, the underlying mechanisms of these cytokines secreted by astrocytes on PMN formation in lung cancer brain metastases remains to be further exploration.

Metabolic disorder is one of the markers of tumor formation, and it also plays a crucial role in the formation of PMN [29]. It is well known that metastatic tumor cells require specific energy, nutrient, and oxygen patterns to compete with the in-situ cells at the site of metastases so that they could adapt to the local tissue microenvironment and establish metastatic colonies. Reports have shown that tumor-cell-derived exosomes could regulate the metabolism of niche cells in specific organs and contribute to PMN formation [30]. Melanoma-derived exosomes alter the metabolic activity of stromal fibroblasts by increasing aerobic glycolysis and decreasing oxidative phosphorylation, therefore leading to extracellular acidification of microenvironment cells at the metastatic site [31].

Fong and his colleagues demonstrated that uptake of miR-122-containing exosomes secreted by breast cancer cells leads to decreased expression of PKM2 and GLUT1 in astrocytes and reduced glucose ingestion [32]. In our study, we used the label-free technique to determine the effect of exosomes secreted by H1299 cells on the protein expression of astrocytes. Functional annotation of differentially expressed proteins, including GO annotation and KEGG annotation, indicated that these differentially expressed proteins were involved in biological processes related to metabolism such as glucose metabolism, amino acid metabolism, and fatty acid metabolism. Therefore, lung cancer cell-derived exosomes could change astrocytes’ protein expression, affect their energy metabolism and accelerate the PMN formation in brain metastasis. The specific internal mechanism remains to be further explored and verified.

## Conclusion

Exosomes produced by H1299 cells could lead to decreased activity and increased apoptosis of astrocytes. They could also stimulate astrocytes to secrete numerous cytokines that promote the formation of the inflammatory and immune inhibitory microenvironment. In addition, the proteomic analysis showed that H1299 cell-derived exosomes changed the expression of the proteins which were mainly involved in regulating metabolic pathways in astrocytes. Overall, lung cancer cell-derived exosomes could promote the formation of PMN in brain metastases by inducing astrocyte apoptosis and affecting cytokine secretion, protein expression, as well as energy metabolism of astrocytes.

## Methods

### Cell Culture

The H1299 cell line was purchased from Zhejiang Meisen Cell Technology Co., Ltd. The cells were cultured in an incubator containing 5% CO2 at 37 °C. When the cell density exceeded 90%, the culture medium was absorbed and discarded. Next, 1 ml trypsin solution was added, and the cells were incubated in the incubator for 3 min until they fell off and separated from the culture bottle wall. Then complete 1640 medium was added to neutralize the trypsin solution. After centrifugation, the cells were re-suspended, and an appropriate amount of re-suspension was placed in a new culture bottle with fresh cell complete medium addition.

### Collection of Cell Supernatant

When the cell density reached more than 90%, the cells were washed twice with PBS, and then the serum-free 1640 medium was added. After 24 hours of cultivation, the cell supernatant was collected into a 50 ml centrifuge tube.

### Extraction of Exosomes from Cell Supernatant

Cell culture supernatant was centrifuged at 2000 g for 30 min to remove cells and debris. After centrifugation, the supernatant was transferred to a new tube, and an appropriate amount of exosome separation reagent was added, shaken, and mixed. The mixed solution was incubated overnight at 2 °C to 8 °C, centrifuged at 4 °C at 10,000 g for 1 hour, and the supernatant was discarded. An appropriate amount of PBS was added to suspend exosomes from the bottom of the centrifuge tube. The extracted exosomes were stored at − 20 degrees.

### NTA Particle Size Detection

The exosomes were diluted to 10^7 / ml-10 ^9 /ml with PBS, and the size and quality of the exosomes were measured by NanoSight NS300 particle potentiometric titration and particle size analyzer.

### Morphology of Exosomes Detected by Transmission Electron Microscope

Ten μl exosomes were dropped on the copper mesh, incubated at room temperature for 10 min, cleaned with sterile distilled water, and absorbent paper was used to absorb excess liquid. Ten μl 2% uranium diacetate was dropped onto the copper mesh and the sample was negatively stained for 1 min. The float was absorbed with filter paper and dried the sample for 2 min under an incandescent lamp. The copper mesh was observed and imaged under the transmission electron microscope.

### Detection of Exosome Protein Markers by Western Blot

CD9, CD63, HSP90, and Alix were used as exosome-positive protein markers, and Calnexin as an exosome-negative protein marker. The separation and concentrated gel were prepared according to the instructions, and 10ul exosomes were added. The gel is put into the prepared electrophoretic solution and 150 V voltage was applied for electrophoresis. PVDF membrane was used for transmembrane. The film transfer device was installed, and the film was transferred on the ice with a constant current of 280 mA and a time of 90 min. After sealing the PVDF membrane for 1 h at room temperature, the primary antibody was added, and the membrane was kept in the refrigerator at 4 °C overnight. After 3 times cleaning in TBST solution, the membrane was incubated with the secondary antibody at room temperature for 1 h. At last, ECL luminous solution was added, and color rendering exposure was carried out by the Chemiluminescence Western Blotting Detection system.

### Detection of the Concentration of Exosomes by BCA Assay

Standard protein and BCA working solution were prepared according to the instructions of BCA kit. Twenty μl standard protein solution was added to 96-well plate with the concentration of 0, 0.025, 0.05, 0.1, 0.2, 0.3, 0.4 and 0.5 mg/ml. Twenty μl exosomes were added into the sample well of the 96-well plate. Two hundred μl BCA working solution was added to each hole and placed at 37 °C for 30 minutes. The absorbance of each hole in the 96-well plate was measured by enzyme-labeled instrument at 562 nm wavelength. The corresponding protein concentration of exosomes was calculated according to the standard curve and the measured absorbance.

### CCK8 Cell Viability Test

The cells were seeded in 96-well plates and incubated until the cell density reached 60%. The medium was replaced by serum-free 1640 medium as well as prepared exosome solution (80 μg/ml, 60 μg/ml, 40 μg/ml, 20 μg/ml). The 96-well plates were placed in an incubator for 24 hours and removed. The medium was discarded and cells were cleaned twice with PBS. CCK8 solution was added according to the instructions and cells were incubated for 1 h. The absorbance of each hole was measured with an enzyme label instrument at 450 nm wavelength, and the cell viability was calculated according to the absorbance.

### Edu/Hoechst 33342 Observation of Cell Apoptosis

The cells of each group were inoculated on 96-well plates. The control group was given serum-free medium, and the experimental group was assigned serum-free medium with H1299 cell-derived exosomes. After incubation for 24 h, the Edu working solution was prepared according to the instructions. After incubating Edu working solution with each group of cells for 2 hours, 4% paraformaldehyde was added for cell fixation at room temperature for 15 minutes. After 3 times washing, the permeable solution was added and incubated for 10 min. Then, the prepared Click reaction solution was added and incubated for 30 min at room temperature under light protection condition. Finally, nuclear staining was performed with Hoechst 33342 and cells were observed under the fluorescence microscope.

### Detection of Apoptosis by Flow Cytometry

The cells were digested with EDTA-free trypsin, centrifuged at 1000 g for 5 min, then re-suspended with 1 ml PBS and counted. 50,000–100,000 cells taken were centrifuged in 1.5 ml EP tubes, and the supernatant was abandoned. Annexin-V-FITC solution was added to re-suspend the cells. Propidium iodide stain was added into each EP tube and the tubes were incubated at room temperature away from light for 20 minutes for detection.

### Detection of Cytokines Secreted by Astrocytes

This experiment was completed in cooperation with Shanghai H Wayne Company Ltd. Bio-Plex Pro Human Cytokine Screening 48-plex Panel chip was used for detection. First, the collected cell supernatant of each group was centrifuged, and the standard sample was prepared according to the instructions. After Assay Buffer was used to dilute and shake microbeads, 50 μl was taken and added to a 96-well plate, followed by 50 μl standard, sample, and Blank, and the samples were incubated at room temperature for 30 min without light. After washing, Detection Antibody was added and incubated for 30 min. Then Streptavidin-PE was added and incubated. After washing, the Assay Buffer was added for shaking and mixing. At last, the Bio-Plex detector was used to detect the concentration of each sample.

### Label-Free Quantitative Proteomics Analysis

This experiment was completed in cooperation with the Shanghai biotechnology corporation. After protein extraction, SDS-PAGE electrophoresis, Coomassie brilliant blue staining, and FASP enzymatic hydrolysis were performed. After chromatographic separation by the Easy nLC system, the samples were analyzed with Orbitrap Exploris 480 mass spectrometer. MaxQuant software (version No. 1.6.14.0) was applied to search the protein database, and LFQ (LabelFreeQuantity) algorithm was used to conduct the protein quantitative analysis. Blast2GO was used to annotate the function of target proteins. KOALA (KEGG Organization and Links Annotation) software was used to annotate the KEGG pathway of the target proteins. During enrichment analysis of GO annotation or KEGG pathway annotation on target proteins, Fisher’s Exact Test was used to compare the distribution of GO classifications or KEGG pathway in target and total proteins to evaluate the significance level of protein enrichment of a certain GO term or KEGG pathway. The software WoLF PSORT was used to predict the location of different proteins.

### Statistical Analysis

The data of flow cytometry were analyzed by Flowjo, and the data of CCK8 were processed by Graphpad Prism 9. The data of different groups were analyzed by unpaired t-test. *P* < 0.05 was considered a statistical difference.

## Data Availability

The date is available from the corresponding author upon reasonable request.

## Abbreviations

BBB: Blood-Brain Barrier
PMN: Pre-Metastatic Niche
CTC: Circulating Tumor Cell
VEGF: Vascular Endothelial Growth Factor
NSCLC: Non-Small Cell Lung Cancer

## References

[1] Cagney DN, Martin AM, Catalano PJ (2017). Incidence and prognosis of patients with brain metastases at diagnosis of systemic malignancy: a population-based study. Neuro-Oncology.

[2] Yamanaka R (2009). Medical management of brain metastases from lung cancer. Oncol Rep.

[3] Deeken JF, Loscher W (2007). The blood-brain barrier and cancer: transporters, treatment, and Trojan horses. Clin Cancer Res.

[4] Wang M, Qin Z, Wan J (2022). Tumor-derived exosomes drive pre-metastatic niche formation in lung via modulating CCL1^+^fibroblast and CCR8^+^ Treg cell interactions. Cancer Immunol Immunother.

[5] Zhuo S, Yang L, Chen S (2022). Ferroptosis: a potential opportunity for intervention of pre-metastatic niche. Front Oncol.

[6] Wang H, Pan J, Barsky L (2021). Characteristics of pre-metastatic niche: the landscape of molecular and cellular pathways. Mol Biomed.

[7] Treps L, Perret R, Edmond S (2017). Glioblastoma stem-like cells secrete the pro-angiogenic VEGF-A factor in extracellular vesicles. J Extracell Vesicles.

[8] Cecchelli R, Berezowski V, Lundquist S (2007). Modelling of the blood-brain barrier in drug discovery and development. Nat Rev Drug Discov.

[9] Arvanitis CD, Ferraro GB, Jain RK (2020). The blood-brain barrier and blood-tumour barrier in brain tumours and metastases. Nat Rev Cancer.

[10] Ye L-Y, Sun L-x, Zhong X-H (2021). The structure of blood-tumor barrier and distribution of chemotherapeutic drugs in non-small cell lung cancer brain metastases. Cancer Cell Int.

[11] Hoshino A, Costa-Silva B, Shen TL (2015). Tumour exosome integrins determine organotropic metastasis. Nature..

[12] Deng J, Liu Y, Lee H (2012). S1PR1-STAT3 signaling is crucial for myeloid cell colonization at future metastatic sites. Cancer Cell.

[13] Orr FW, Wang HH, Lafrenie RM (2000). Interactions between cancer cells and the endothelium in metastasis. J Pathol.

[14] Tominaga N, Kosaka N, Ono M, et al. Brain metastatic cancer cells release microRNA-181c-containing extracellular vesicles capable of destructing blood-brain barrier. Nat Commun. 2015;6:6716. 10.1038/ncomms7716 PMC4396394 25828099

[15] Kinjyo I, Bragin D, Grattan R (2019). Leukemia-derived exosomes and cytokines pave the way for entry into the brain. J Leukoc Biol.

[16] Liu Y, Cao X (2016). Characteristics and significance of the pre-metastatic niche. Cancer Cell.

[17] Guo Y, Ji X, Liu J (2019). Effects of exosomes on premetastatic niche formation in tumors. Mol Cancer.

[18] Lobb RJ, Lima LG, Möller A (2017). Exosomes: key mediators of metastasis and premetastatic niche formation. Semin Cell Dev Biol.

[19] Valiente M, Obenauf AC, Jin X (2014). Serpins promote cancer cell survival and vascular co-option in brain metastasis. Cell..

[20] Klein A, Schwartz H, Sagi-Assif O (2015). Astrocytes facilitate melanoma brain metastasis via secretion of IL-23. J Pathol.

[21] Xing F, Liu Y, Sharma S (2016). Activation of the cMet pathway mobilizes an inflammatory network in the brain microenvironment to promote brain metastasis of breast cancer. Cancer Res.

[22] Chen Q, Boire A, Jin X (2016). Carcinoma-astrocyte gap junctions promote brain metastasis by cGAMP transfer. Nature..

[23] Lukanidin E, Sleeman JP (2012). Building the niche: the role of the S100 proteins in metastatic growth. Semin Cancer Biol.

[24] Bresnick AR, Weber DJ, Zimmer DB (2015). S100 proteins in cancer. Nat Rev Cancer.

[25] Zhou Y, Zhang J, Liu Q (2005). The chemokine GRO-alpha (CXCL1) confers increased tumorigenicity to glioma cells. Carcinogenesis..

[26] Abbott NJ (2002). Astrocyte-endothelial interactions and blood-brain barrier permeability. J Anat.

[27] Deng Z, Zhou L, Wang Y (2020). Astrocyte-derived VEGF increases cerebral microvascular permeability under high salt conditions. Aging (Albany NY).

[28] Kremlev SG, Gaurnier-Hausser AL, Del Valle L (2008). Angiocidin promotes pro-inflammatory cytokine production and antigen presentation in multiple sclerosis. J Neuroimmunol.

[29] Loo JM, Scherl A, Nguyen A (2015). Extracellular metabolic energetics can promote cancer progression. Cell..

[30] Morrissey SM, Zhang F, Ding C (2021). Tumor-derived exosomes drive immunosuppressive macrophages in a pre-metastatic niche through glycolytic dominant metabolic reprogramming. Cell Metab.

[31] Shu S, Yang Y, Allen CL (2018). Metabolic reprogramming of stromal fibroblasts by melanoma exosome microRNA favours a pre-metastatic microenvironment. Sci Rep.

[32] Fong MY, Zhou W, Liu L (2015). Breast-cancer-secreted miR-122 reprograms glucose metabolism in premetastatic niche to promote metastasis. Nat Cell Biol.

